# Gout increases risk of fracture: A nationwide population-based cohort study

**DOI:** 10.1097/MD.0000000000004669

**Published:** 2016-08-26

**Authors:** Huey-En Tzeng, Che-Chen Lin, I-Kuan Wang, Po-Hao Huang, Chun-Hao Tsai

**Affiliations:** a Graduate Institute of Clinical Medicine; b School of Medicine, China Medical University; c Division of Hematology/Oncology; d Healthcare Service Research Center (HSRC), Taichung Veterans General Hospital; e Division of Nephrology; f Division of Rheumatology; g Department of Orthopedic Surgery, China Medical University Hospital, Taichung, Taiwan.

**Keywords:** fracture, gout, retrospective cohort study, risk

## Abstract

There is still debate on whether high uric acid increases bone mineral density (BMD) against osteoporotic fracture or bone resorption caused by gout inflammation. This study aimed to evaluate whether gout offers a protective effect on bone health or not. We conducted a nationwide population-based retrospective cohort study to evaluate the association between gout history and risk factors of fracture.

A retrospective cohort study was designed using the claim data from Longitudinal Health Insurance Database (LHID). A total of 43,647 subjects with gout and a cohort of 87,294 comparison subjects without gout were matched in terms of age and sex between 2001 and 2009, and the data were followed until December 31, 2011. The primary outcome of the study was the fracture incidence, and the impacts of gout on fracture risks were analyzed using the Cox proportional hazards model.

After an 11-year follow-up period, 6992 and 11,412 incidents of fracture were reported in gout and comparison cohorts, respectively. The overall incidence rate of fracture in individuals with gout was nearly 23%, which was higher than that in individuals without gout (252 vs 205 per 10,000 person-years) at an adjusted hazard ratio of 1.17 (95% confidence interval = 1.14–1.21). Age, sex, and fracture-associated comorbidities were adjusted accordingly. As for fracture locations, patients with gout were found at significant higher fracture risks for upper/lower limbs and spine fractures. In gout patient, the user of allopurinol or benzbromarone has significantly lower risk of facture than nonusers.

Gout history is considered as a risk factor for fractures, particularly in female individuals and fracture sites located at the spine or upper/lower limbs.

## 1 Introduction

Gout, a monosodium urate crystal deposition disease in various parts of the body, is the most prevalent form of inflammatory arthritis worldwide, causing substantial morbidity.^[1,2]^ Gout can be characterized biochemically using extracellular fluid urate saturation, that is, gout crystal forms and the precipitation increases once urate concentration exceeds 380 μmol/L. Hyperuricemia is a risk factor for gout, but gout can still occur when serum uric acid (UA) levels are low.^[3]^

There are literatures debating that serum UA levels are associated with bone health. Mehta et al^[4]^ reported that increased serum urate levels were associated with an increased risk of hip fractures in men. However, others showed that UA was a protective factor against the development of incident osteoporotic fractures (OFs) in men.^[5]^ In addition, higher serum UA levels were negatively proportion to the incidence of non-spine fractures except hip fractures.^[6]^

Hyperuricemia, which is different from gout, is a necessary but not sufficient predisposing factor for the development of urate crystal deposition disease. Because the protective effects of hyperuricemia on bone mineral density (BMD) are still uncertain, the comorbidities between gout cohorts with or without osteoporosis should be further analyzed. The prevalence of gout increased with age is in association with the primary risk factors of fracture. In summary, gout-induced poor bone health and increased risks of fracture are hence of great public health importance, particularly to the elderly. However, to our knowledge, literatures regarding gout, and bone health or fracture risks are still missing.

The aim of this study was to evaluate whether gout comprised protective effects on risk factors of bony fracture. We conducted a nationwide population-based retrospective cohort study and used the database from a universal insurance program to evaluate the association between gout history and risk factors of fracture.

## 2 Methods

### 2.1 Data source

The Longitudinal Health Insurance Database (LHID), which established by National Health Research Institutes (NHRI) in Taiwan, collected data from people covered with Taiwan National Health Insurance program (Taiwan NHI). Data collected for LHID were randomly selected and followed from a cohort of 1 million people covered by the national insurance program between 1996 and 2000. No statistical significance was found in the distributions of age and sex between the cohort in LHID and the Taiwan NHI enrollees. LHID involves claim data from Taiwan NHI, including registry of beneficiary, registry of prescription, registry of clinical visits, and hospital care as well as other medical services. The disease code recorded in the registry of clinical visits and hospital care was based on the International Classification of Diseases, Ninth Revision, Clinical Modification (ICD-9-CM). To protect the privacy of people involved in the database, NHRI only released data with encoded identification numbers, so personnel without authorization would not be able to reveal or link any direct information to the enrollees. In addition, this study was approved by the Ethics Review Board of China Medical University (CMUH104-REC2-115).

### 2.2 Study population

To evaluate the prevalence of fracture in patients with gout, we selected a cohort of patients with gout and a comparable control cohort of normal subjects (without gout). The cohort of patients with gout (ICD-9-CM 274) was diagnosed between January 1, 2001 and December 31, 2009, and the age of definitive diagnosis was set >20 years old. The index dates of patients with gout were the dates of first definitive diagnoses. The comparison cohort was randomly selected from LHID enrollees without any histories of gouts, and the selection frequency was at 1:2 ratios of age and sex. Subjects in the comparison cohort and paired with matching cases were randomly assigned a month and day in the same year. Subjects that had a history of fractures before index date were excluded from both study cohorts. The end point was the incidence of fractures on the date of subject withdrawal or the end of the follow-up (December 31, 2011).

The primary outcome of the study was the individual event of the fracture (ICD-9-CM 800-829), and the hazard ratios of various types, including hip fractures (ICD-9-CM 820), vertebral fractures (ICD-9-CM 805), wrist fractures (ICD-9-CM 815), proximal humerus fractures (ICD-9-CM 812.0 and 812.1), other upper limb fractures (ICD-9-CM 810, 812-813, except for 812.0 and 812.1), and fractures of the thigh/leg/ankle (ICD-9-CM 821, 823–825) between the cohorts of gout and comparison group were also of great interests.

Confounding factors such as age, sex, and comorbidity were adjusted accordingly. The individual with comorbidity was the study subject with the history of comorbidity before the index date. The comorbidities included alcohol-related disorder (ALD, ICD-9-CM 291, 303, 305, 571.0, 571.1, 571.2, 571.3, 790.3, and V11.3), diabetes mellitus (DM, ICD-9-CM 250), hypertension (ICD-9-CM 401-405), osteoporosis (ICD-9-CM 733.0 and 733.1), stroke (ICD-9-CM 430-438), Parkinson disease (ICD-9-CM 332), chronic obstructive pulmonary disease (COPD, ICD-9-CM 491, 492, and 496), coronary artery disease (CAD, ICD-9-CM 410-414), and end-stage renal disease (ICD-9-CM 585 from catastrophic illness files). This study also considered the effect of anti-gout drug for risk of fracture in gout patient and listed the drug, including allopurinol (the Anatomical Therapeutic Chemical [ATC] code M04AA01) and benzbromarone (ATC code M04AB03). To standardize the drug exposure, we transformed to defined daily dose (DDD) based on ATC definition.

### 2.3 Statistical analysis

The dichotomous variables, presented as number and percentage, included sex (male/female) and comorbidities (no/yes). Continuous variables, such as age, were showed as mean and standard deviation (SD) and analyzed using an independent *t* test. Nominal parameters such as sex and comorbidity were assessed using a Chi-square test. Kaplan–Meier method was applied to calculate the incidence densities of subsequent fractures and the cumulative incidence curves between gout cohort and comparison cohort. The differences of incidence curves between these 2 cohorts were analyzed using a log rank test. To investigate the associations between fracture and gout, the hazard ratios (HRs) and 95% confidence intervals (CIs) for patients with gout in comparison with subjects in the comparison cohort were estimated using crude and adjusted Cox proportional hazard models. SAS 9.4 software (SAS Institute, Cary, NC) was used to perform the analyses and R software (R Foundation for Statistical computing, Vienna, Austria) was used to figure the incidence curves. The significant level was set at less than 0.05 for 2-side testing.

## 3 Results

This study involved 43,647 patients with gout and 2-fold corresponding amount of comparison cohort (Table 1). Because this was an age- and sex-matched study, the mean age was 50.9 years old (SD = 16.0) and the percentage of male subjects was 69.9%. The proportion of ESRD and Parkinson disease was lower among patients with gout than those among compassion cohort, but other comorbidities were significantly prevalent in the cohort of gout.

**Table 1:**
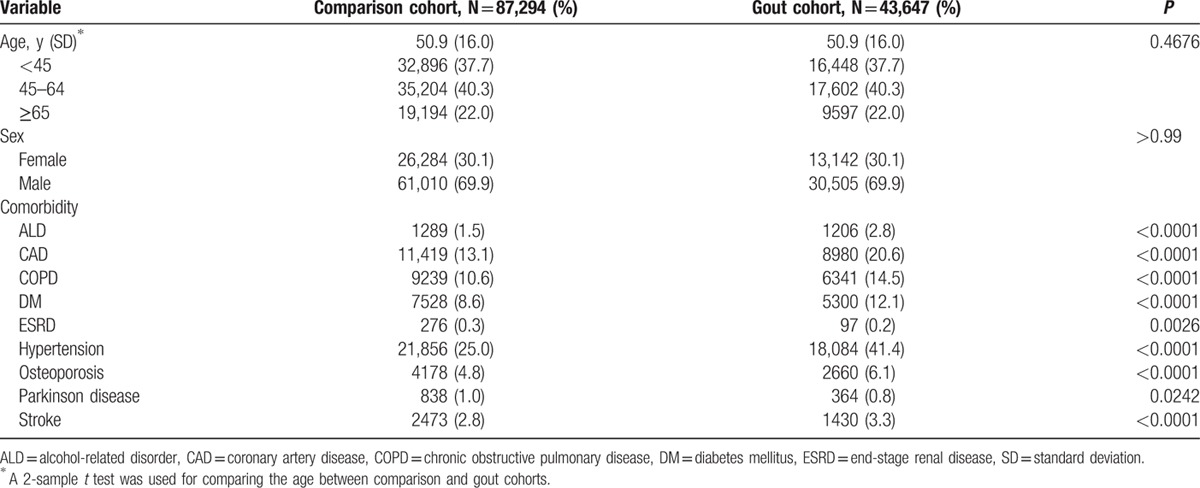
Baseline demographics and comorbidity between comparison and gout cohorts.

**Figure 1:**
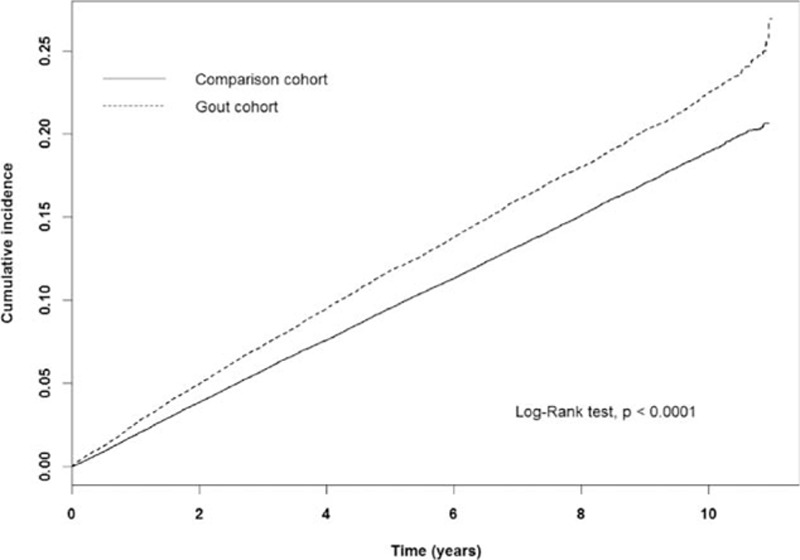
Cumulative incidence of developing fracture in comparison and gout cohort.

In this study, a total of 18,404 participants developed fracture events (Table 2). The incidence of fractures was nearly 1.23-fold greater than that of comparison cohort (252 vs 205 per 10,000 person-years). The cumulative incidence curve of fracture was significant higher in gout cohort in comparison with control cohort (Fig. 1) (log rank test, *P* < 0.0001). Individuals with increased age or a history of comorbidities were predisposing factors for fractures, and males had a lower risk of fractures than that of females. After adjusting age, sex, and comorbidities, the gout cohort had a 1.17-fold increased risk of developing fracture than those without fractures (HR = 1.17, 95% CI = 1.14–1.21).

**Table 2:**
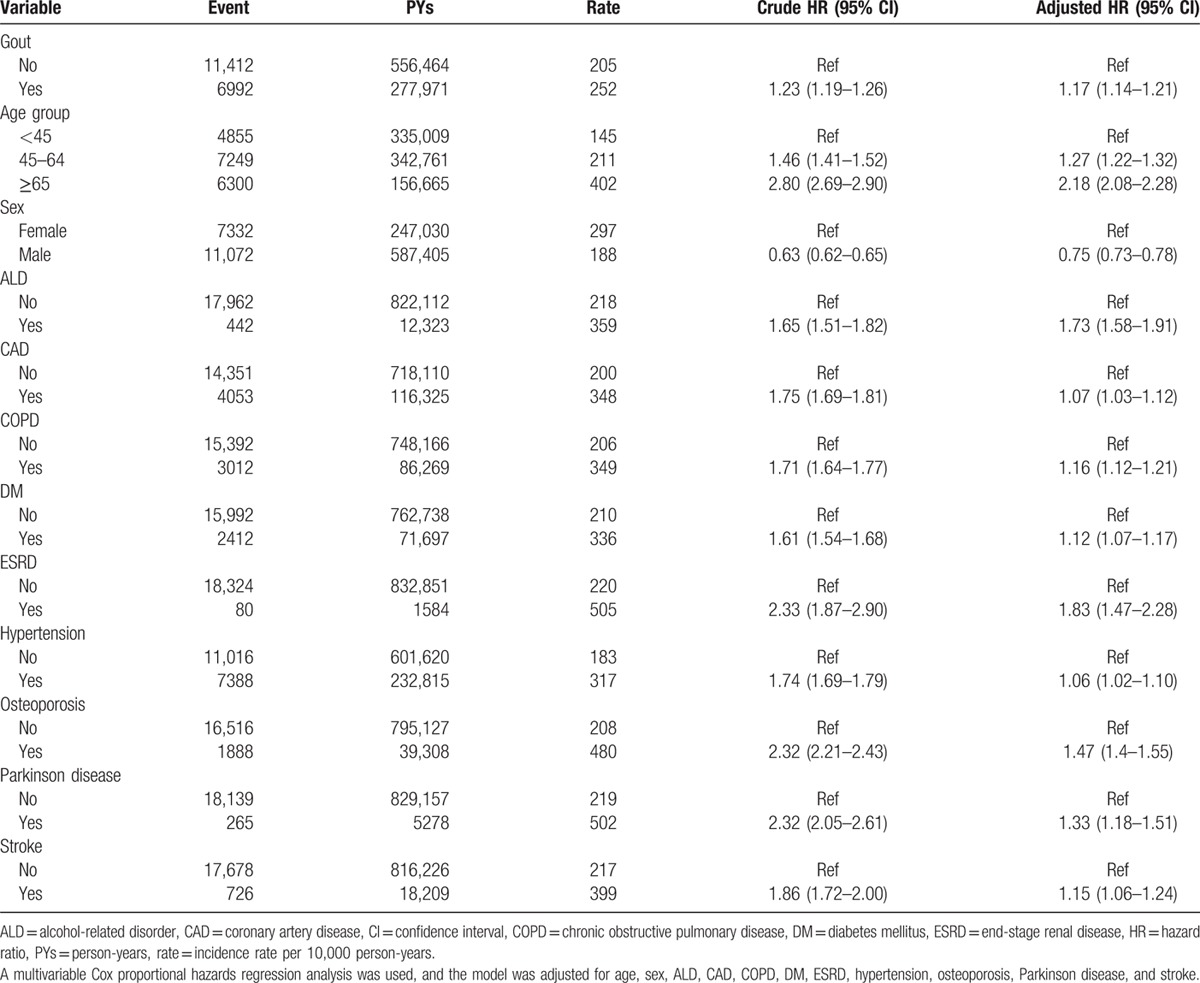
Incidence of fracture and corresponding hazard ratio for study cohort.

Table 3 shows the risks of fractures depending on locations between gout and comparison cohorts. Relative to the comparison cohort, the significant higher risk of vertebral fracture, other upper limb fracture, leg/knee fracture, and ankle/foot fracture were 1.14-fold (95% CI = 1.05–1.23), 1.08-fold (95% CI = 1.01–1.16), 1.19-fold (95% CI = 1.07–1.32), and 1.34-fold (95% CI = 1.24–1.45) for gout cohort, respectively. On the contrary, no statistical significances of increased risks were found in hip fractures (HR = 0.97, 95% CI = 0.86–1.09), wrist fractures (HR = 1.19, 95% CI = 1.00–1.43), proximal humerus fractures (HR = 1.15, 95% CI = 0.95–1.38), or thigh fracture (HR = 1.01, 95% CI = 0.83–1.24) between gout and comparison cohorts. With comparison cohorts, the gout cohort still had a significantly higher risk of vertebral fractures (HR = 1.16, 95% CI = 1.06–1.26), other upper limb fractures (HR = 1.09, 95% CI = 1.01–1.17), leg/knee fracture (HR = 1.19, 95% CI = 1.06–1.33), or ankle/foot (HR = 1.36, 95% CI = 1.25–1.48) even without having any histories of osteoporosis. However, no significant differences of risks for all subtypes of fractures were found between gout and comparison cohorts with the history of osteoporosis.

**Table 3:**
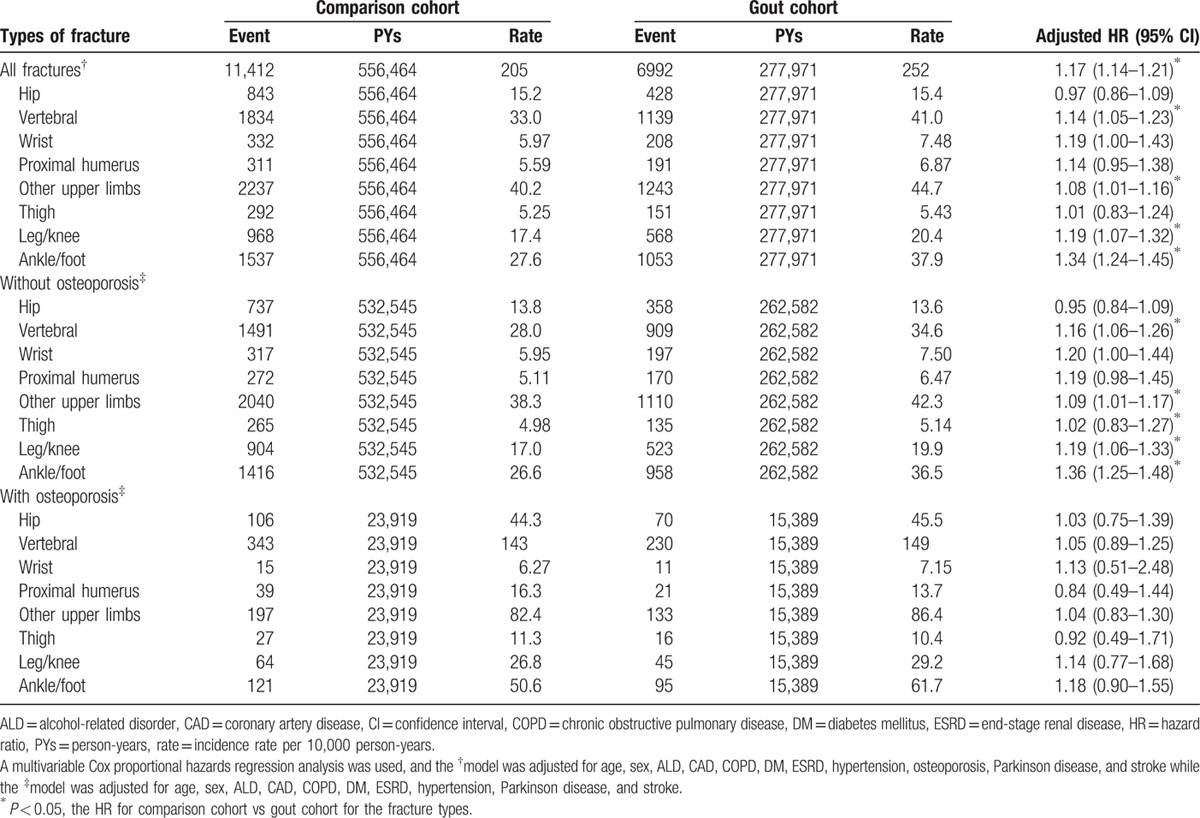
Incidence of fracture types and hazard ratios for study cohort.

Table 4 shows the risks of fracture following the average frequencies of gout visits. Relative to the comparison cohort, the HRs of fractures among patients with gout at average visiting frequency <3, 3 to 8, and ≥9 times were 1.00 (95% CI = 0.97–1.03), 1.73 (95% CI = 1.63–1.83), and 5.97 (95% CI = 5.49–6.50), respectively. A significantly increased trend of fracture risk along with increased average times of gout visits was also found (*P* for trend <0.0001).

**Table 4:**
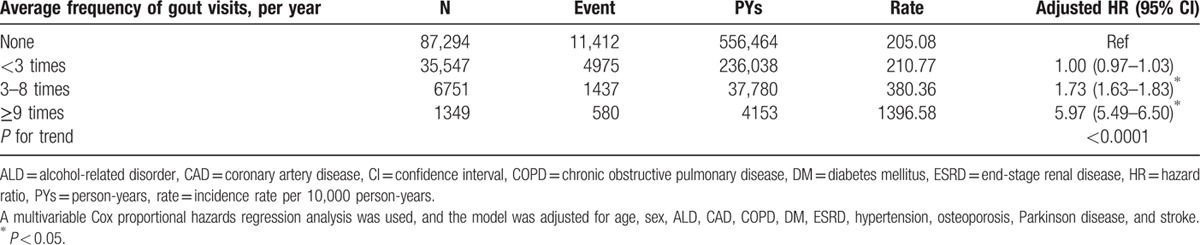
Incidence of fracture and hazard ratios for study cohort between different levels of gout visits.

Table 5 shows the risk of fracture among the gout patients receiving different antigout drugs. After adjusted for age, sex, comorbidity, and each anti-gout drugs, the HRs of fracture risk were 0.72 (95% CI = 0.67–0.78) and 0.71 (95% CI = 0.68–0.75) for allopurinol users and benzbromarone users, respectively. Both drug also revealed a decreased trend of fracture risk followed by an increasing allopurinol exposure (*P* for trend <0.0001) and benzbromarone exposure (*P* for trend <0.0001).

**Table 5:**
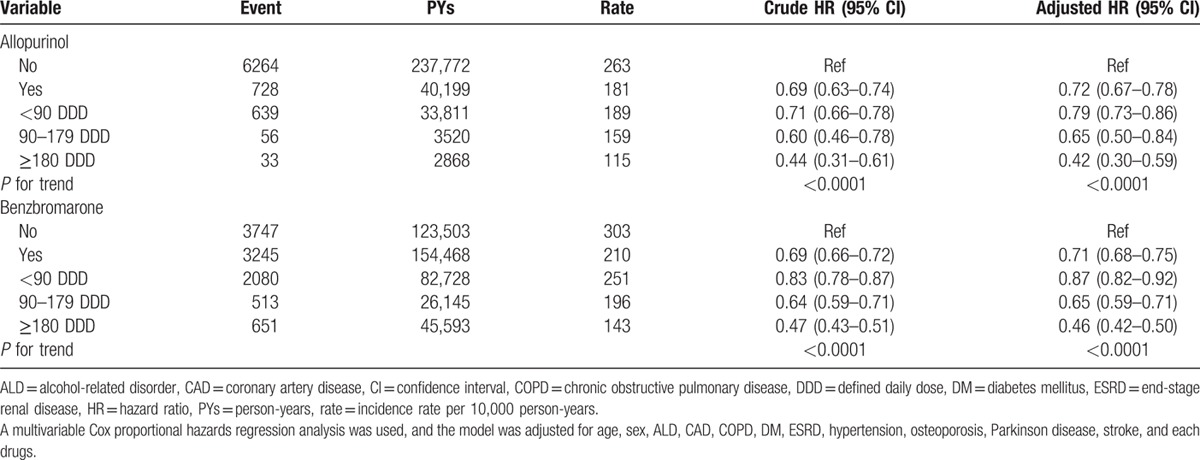
Incidence of fracture and multivariate Cox proportional hazards regression analysis measured hazard ratio for different antigout drugs in gout patients.

## 4 Discussion

The present study demonstrated an increased risk of fracture resulted from the history of gout, particularly in female individuals. Our result also revealed the risk of fracture in gout cohort with/without the presence of osteoporosis. In cases without osteoporosis, gout only increased the risk of fracture in spine and lower limbs, but those were insignificant in cases with osteoporosis. In cases with gout, patients receiving antigout drugs, allopurinol and benzbromarone, had lower risk of fracture. The cause–effect relationship between gout attack and the increased risks of fracture may be explained using inflammation-induced bone loss or accident hypotheses resulted from acute or chronic gout arthropathy.

Circulating inflammatory markers may be related to the changes in and the resorption of BMD among older adults. Zheng et al^[7]^ reported that stimulated peripheral monocyte blood cells may be responsible for the correlations between the secretion of IL-1β, IL-6, and TNF-α and the BMD of lumbar spine. Polzer et al^[8]^ also presented that interleukin-1 is essential for systemic inflammatory bone loss. While tophi formed, monocytes may contact with the crystals of monosodium urate monohydrate (MSU) and then phagocytosed by PMNs. IL-1β/IL-1R signaling is essential for MSU-induced inflammation.^[9]^ Macrophage- or monocyte-released IL-1 within the joint may induce the expression of adhesion molecules, such as E-selectin and chemokines (e.g., CXCL8) to recruit other cells (e.g., neutrophils, endothelial cells, and fibroblasts) to the joint.^[9]^ The inflammatory cascades during gout attack may produce increased levels of proinflammatory cytokines such as IL-1β, and then leads to bone resorption. IL-1β is a strong stimulator of in vitro and in vivo bone resorption.^[10–12]^ IL-1β not only upregulates the production of RANKL and enhances its activity, but also stimulates osteoclastogenesis.^[13]^ The activation of RANKL-RANK induces the expression of c-Fos, and subsequently promotes nuclear translocation of Jun proteins and NFATc1.^[14,15]^ These effects further promote osteoclast differentiation, activation, survival, and thus leading to bone resorption.^[16]^ In addition, IL-1β increases prostaglandin synthesis in bone,^[10,17]^ which displays a potent resorption stimulus.^[10]^ Moreover, IL-1β strongly decreased new bone formation by upregulating Dickkopf-related protein 1 (DKK1) and sclerostin (SOST), which may downregulate osteoblasts.^[17]^

In addition, uric acid itself has an impact on oxidative stress. Uric acid is a product of purine nucleotides catabolism, which can be catalyzed by liver enzymes such as xanthine oxidoreductase (XOR). XOR enables the oxidation of hypoxanthine. As a result, xanthine can be further oxidized to uric acid.^[18]^ During the production of uric acid, which can be catalyzed by xanthine oxidase, excessive free radicals, and reactive oxygen species (ROS) are also generated as by-products. These ROS stimulate osteoclast differentiation and bone resorption in murine calvarial and bone marrow cultures as well as in osteoblasts/spleen cells cocultures by the stimulation of the osteoclastogenic nuclear factor-kappa β ligand (RANKL)–RANK signaling pathway.^[19]^ Binding of RANKL to RANK initiates osteoclast differentiation/activation, and thus is critical for their survival and promotion of bone resorption, and ultimately plays a role in pathogenesis of postmenopausal osteoporosis.^[20,21]^

Gout is more common in men than in women, but women become increasingly prevalent to gout after menopause. The increase level of ROS also explained hyperuricemia parallel with the decrease of estrogen levels.^[22]^ Estrogens have systemic antioxidant effects,^[23,24]^ and the protective effects on bones, especially E2, are deficient in menopausal status.^[25]^

Study has proved that higher uric acid levels within the physiological range may be beneficial to BMD.^[26]^ However, our data showed that gout itself increased the risks of fractures in lower limbs and spine, even in cases without osteoporosis. Dennison et al^[27]^ presented that a very high urate level was associated with an acute, intense inflammatory arthritis, which was not beneficial to bone health. Gouty arthritis requiring allopurinol is associated with an excess risk of major or hip fracture.^[27]^ Because gout most occurs over the lower limbs, the functions of lower limbs may be impaired and the risks of fracture may also be increased accordingly.^[27]^ Even subjects did not meet the criteria of osteoporosis, gout inflammation may result in increased risks of spine fracture through bone resorption.

Our results revealed the importance of gout prevention and treatment for subjects with osteoporosis. Gout attacks are like glucosteroids, which can be used as a common therapy for inflammatory disease. There is no doubt about the deleterious effect of GC in bone metabolism by suppressing bone formation and enhancing bone resorption and thus increases the risks of fracture.^[28–30]^ Therefore, the assessment of BMD or identify the risks of fragile fracture in patients with gout, especially in the elderly and female populations is warrant. Interleukin-1 antagonists could be an alternative option in gout, even though the BMD of these patients may not meet the criteria of osteoporosis. IL-1 blockers have well proved its efficacy on the treatment of acute gouty arthritis or for the prevention of gout flares.^[31–44]^ Baltzer et al^[45]^ reported that the transfer of the IL-1Ra gene strongly reduced the early loss of bone mass occurring in responses to ovariectomy. However, no literatures regarding the changes in BMD or fracture risks after the use of IL-1 blockers were mentioned. Antioxidant supplements such as vitamin D and vitamin E can also play a role in preventing inflammation and osteoporosis in patients with gout. Vitamin D not only prevents bone resorption, but also has immune-modulatory and in vitro antiinflammatory properties.^[46–48]^ Vitamin E, a potent antioxidant, is able to neutralize free radicals and inhibits COX-2; therefore may be able to suppress cytokine production and osteoporosis.^[49]^ Among the 2 types of vitamin E, tocotrienolis is better than tocopherol due to the ability to suppress bone resorbing cytokines.^[50]^

The strengths of this study included the use of population-based data that were highly representative of a general population. However, several factors limited this study. First, the National Health Insurance research database (NHIRD) does not contain detailed information regarding BMI, smoking habit, alcohol consumption, BMD, and serum uric acid data, which may be considered as relevant data involving risks of fracture. Second, our study proved a decreased trend of fracture risk followed by the use of allopurinol or benzbromarone, which reflects that regular gout control decrease risk of fracture. But we could not assess other novel medication, such as IL-1 inhibitor due to not current indication in gout therapy by Taiwan NHI regulation. Third, the evidence derived from a retrospective cohort study was generally lower in statistical power than that from randomized controlled trials because of potential bias related to confounding variables adjustments. However, this analysis derived from a very large, well-represented data set, which provided a clear assessment of fracture among patients with gouty arthritis. Therefore, further studies using osteoporosis as another outcome measure is needed.

## 5 Conclusions

While there is still debate on the protective effects of high uric acid on BMD, our study revealed that gout increased the overall risks of bony fractures, especially for females and those with fractures in the spine/lower legs. The treatment of gout should include fracture evaluation, prevention, and antioxidant supplementation as well as different classes of drugs that are approved for gout and osteoporotic prevention, including further medications such as interleukin-1 antagonists.

## References

[1] Lee SJ Hirsch JD Terkeltaub R Perceptions of disease and health-related quality of life among patients with gout. *Rheumatology (Oxford)* 2009; 48:582–586. 19307257 10.1093/rheumatology/kep047 PMC2722803

[2] Halabe A Sperling O Uric acid nephrolithiasis. *Miner Electrolyte Metab* 1994; 20:424–431. 7783706

[3] Liebman SE Taylor JG Bushinsky DA Uric acid nephrolithiasis. *Curr Rheumatol Rep* 2007; 9:251–257. 17531180 10.1007/s11926-007-0040-z

[4] Mehta T Buzkova P Sarnak MJ Serum urate levels and the risk of hip fractures: data from the Cardiovascular Health Study. *Metabolism* 2015; 64:438–446. 25491429 10.1016/j.metabol.2014.11.006 PMC4312534

[5] Kim BJ Baek S Ahn SH Higher serum uric acid as a protective factor against incident osteoporotic fractures in Korean men: a longitudinal study using the National Claim Registry. *Osteoporos Int* 2014; 25:1837–1844. 24668006 10.1007/s00198-014-2697-2

[6] Lane NE Parimi N Lui LY Association of serum uric acid and incident nonspine fractures in elderly men: the Osteoporotic Fractures in Men (MrOS) study. *J Bone Miner Res* 2014; 29:1701–1707. 24347506 10.1002/jbmr.2164 PMC4351860

[7] Zheng SX Vrindts Y Lopez M Increase in cytokine production (IL-1 beta, IL-6, TNF-alpha but not IFN-gamma, GM-CSF or LIF) by stimulated whole blood cells in postmenopausal osteoporosis. *Maturitas* 1997; 26:63–71. 9032749 10.1016/s0378-5122(96)01080-8

[8] Polzer K Joosten L Gasser J Interleukin-1 is essential for systemic inflammatory bone loss. *Ann Rheum Dis* 2010; 69:284–290. 19196726 10.1136/ard.2008.104786

[9] Chen CJ Shi Y Hearn A MyD88-dependent IL-1 receptor signaling is essential for gouty inflammation stimulated by monosodium urate crystals. *J Clin Invest* 2006; 116:2262–2271. 16886064 10.1172/JCI28075 PMC1523415

[10] Lorenzo J Horowitz M Choi Y Osteoimmunology: interactions of the bone and immune system. *Endocr Rev* 2008; 29:403–440. 18451259 10.1210/er.2007-0038 PMC2528852

[11] Garlanda C Dinarello CA Mantovani A The interleukin-1 family: back to the future. *Immunity* 2013; 39:1003–1018. 24332029 10.1016/j.immuni.2013.11.010 PMC3933951

[12] Dinarello CA Overview of the interleukin-1 family of ligands and receptors. *Semin Immunol* 2013; 25:389–393. 24275600 10.1016/j.smim.2013.10.001

[13] Nakamura I Jimi E Regulation of osteoclast differentiation and function by interleukin-1. *Vitam Horm* 2006; 74:357–370. 17027523 10.1016/S0083-6729(06)74015-8

[14] Lu X Gilbert L He X Transcriptional regulation of the osterix (Osx, Sp7) promoter by tumor necrosis factor identifies disparate effects of mitogen-activated protein kinase and NF kappa B pathways. *J Biol Chem* 2006; 281:6297–6306. 16410254 10.1074/jbc.M507804200

[15] Diarra D Stolina M Polzer K Dickkopf-1 is a master regulator of joint remodeling. *Nat Med* 2007; 13:156–163. 17237793 10.1038/nm1538

[16] Corrado A Neve A Maruotti N Bone effects of biologic drugs in rheumatoid arthritis. *Clin Dev Immunol* 2013; 2013:945945. 23864880 10.1155/2013/945945 PMC3705836

[17] Redlich K Smolen JS Inflammatory bone loss: pathogenesis and therapeutic intervention. *Nat Rev Drug Discov* 2012; 11:234–250. 22378270 10.1038/nrd3669

[18] Riegersperger M Covic A Goldsmith D Allopurinol, uric acid, and oxidative stress in cardiorenal disease. *Int Urol Nephrol* 2011; 43:441–449. 21547469 10.1007/s11255-011-9929-6

[19] Baek KH Oh KW Lee WY Association of oxidative stress with postmenopausal osteoporosis and the effects of hydrogen peroxide on osteoclast formation in human bone marrow cell cultures. *Calcif Tissue Int* 2010; 87:226–235. 20614110 10.1007/s00223-010-9393-9

[20] Vega D Maalouf NM Sakhaee K Clinical review #: the role of receptor activator of nuclear factor-kappaB (RANK)/RANK ligand/osteoprotegerin: clinical implications. *J Clin Endocrinol Metab* 2007; 92:4514–4521. 17895323 10.1210/jc.2007-0646

[21] Ozgocmen S Kaya H Fadillioglu E Role of antioxidant systems, lipid peroxidation, and nitric oxide in postmenopausal osteoporosis. *Mol Cell Biochem* 2007; 295:45–52. 16841180 10.1007/s11010-006-9270-z

[22] Hak AE Choi HK Menopause, postmenopausal hormone use and serum uric acid levels in US women-the Third National Health and Nutrition Examination Survey. *Arthritis Res Ther* 2008; 10:R116. 18822120 10.1186/ar2519 PMC2592803

[23] Watanobe H Schioth HB Nitric oxide mediates leptin-induced preovulatory luteinizing hormone and prolactin surges in rats. *Brain Res* 2001; 923:193–197. 11743988 10.1016/s0006-8993(01)03247-4

[24] Priyanka HP Singh RV Pratap UP Estrogen modulates beta2-adrenoceptor-induced cell-mediated and inflammatory immune responses through ER-alpha involving distinct intracellular signaling pathways, antioxidant enzymes, and nitric oxide. *Cell Immunol* 2014; 292:1–8. 25240148 10.1016/j.cellimm.2014.08.001

[25] Horowitz MC Cytokines and estrogen in bone: anti-osteoporotic effects. *Science* 1993; 260:626–627. 8480174 10.1126/science.8480174

[26] Dalbeth N Topless R Flynn T Mendelian randomization analysis to examine for a causal effect of urate on bone mineral density. *J Bone Miner Res* 2015; 30:985–991. 25502344 10.1002/jbmr.2434

[27] Dennison EM Rubin KH Schwarz P Is allopurinol use associated with an excess risk of osteoporotic fracture? A National Prescription Registry study. *Arch Osteoporos* 2015; 10:36. 26481934 10.1007/s11657-015-0241-4 PMC5384630

[28] Yongtao Z Kunzheng W Jingjing Z Glucocorticoids activate the local renin-angiotensin system in bone: possible mechanism for glucocorticoid-induced osteoporosis. *Endocrine* 2014; 47:598–608. 24519760 10.1007/s12020-014-0196-z

[29] Ohnaka K Taniguchi H Kawate H Glucocorticoid enhances the expression of dickkopf-1 in human osteoblasts: novel mechanism of glucocorticoid-induced osteoporosis. *Biochem Biophys Res Commun* 2004; 318:259–264. 15110782 10.1016/j.bbrc.2004.04.025

[30] Sasaki N Kusano E Ando Y Glucocorticoid decreases circulating osteoprotegerin (OPG): possible mechanism for glucocorticoid induced osteoporosis. *Nephrol Dial Transplant* 2001; 16:479–482. 11239019 10.1093/ndt/16.3.479

[31] Tran AP Edelman J Interleukin-1 inhibition by anakinra in refractory chronic tophaceous gout. *Int J Rheum Dis* 2011; 14:e33–e37. 21816011 10.1111/j.1756-185X.2011.01629.x

[32] Singh D Huston KK IL-1 inhibition with anakinra in a patient with refractory gout. *J Clin Rheumatol* 2009; 15:366. 20009976 10.1097/RHU.0b013e3181be2423

[33] Funck-Brentano T Salliot C Leboime A First observation of the efficacy of IL-1ra to treat tophaceous gout of the lumbar spine. *Rheumatology (Oxford)* 2011; 50:622–624. 21097448 10.1093/rheumatology/keq358

[34] Chen K Fields T Mancuso CA Anakinra's efficacy is variable in refractory gout: report of ten cases. *Semin Arthritis Rheum* 2010; 40:210–214. 20494407 10.1016/j.semarthrit.2010.03.001

[35] Tran TH Pham JT Shafeeq H Role of interleukin-1 inhibitors in the management of gout. *Pharmacotherapy* 2013; 33:744–753. 23553601 10.1002/phar.1265

[36] Schlesinger N Anti-interleukin-1 therapy in the management of gout. *Curr Rheumatol Rep* 2014; 16:398. 24407823 10.1007/s11926-013-0398-z

[37] Ottaviani S Molto A Ea HK Efficacy of anakinra in gouty arthritis: a retrospective study of 40 cases. *Arthritis Res Ther* 2013; 15:R123. 24432362 10.1186/ar4303 PMC3978950

[38] Molto A Ea HK Richette P Efficacy of anakinra for refractory acute calcium pyrophosphate crystal arthritis. *Joint Bone Spine* 2012; 79:621–623. 22658375 10.1016/j.jbspin.2012.01.010

[39] Jesus AA Goldbach-Mansky R IL-1 blockade in autoinflammatory syndromes. *Annu Rev Med* 2014; 65:223–244. 24422572 10.1146/annurev-med-061512-150641 PMC4178953

[40] Ghosh P Cho M Rawat G Treatment of acute gouty arthritis in complex hospitalized patients with anakinra. *Arthritis Care Res (Hoboken)* 2013; 65:1381–1384. 23650178 10.1002/acr.21989

[41] Dumusc A So A Interleukin-1 as a therapeutic target in gout. *Curr Opin Rheumatol* 2015; 27:156–163. 25633244 10.1097/BOR.0000000000000143

[42] Dinarello CA van der Meer JW Treating inflammation by blocking interleukin-1 in humans. *Semin Immunol* 2013; 25:469–484. 24275598 10.1016/j.smim.2013.10.008 PMC3953875

[43] Bartov JB Ali Y Successful use of the interleukin 1 antagonist, anakinra, in a patient with gout, chronic kidney disease, and aplastic anemia. *J Clin Rheumatol* 2013; 19:454–456. 24263151 10.1097/RHU.0000000000000047

[44] Aouba A Deshayes S Frenzel L Efficacy of anakinra for various types of crystal-induced arthritis in complex hospitalized patients: a case series and review of the literature. *Mediators Inflamm* 2015; 2015:792173. 25922564 10.1155/2015/792173 PMC4398911

[45] Baltzer AW Whalen JD Wooley P Gene therapy for osteoporosis: evaluation in a murine ovariectomy model. *Gene Ther* 2001; 8:1770–1776. 11803396 10.1038/sj.gt.3301594

[46] Amer M Qayyum R Relation between serum 25-hydroxyvitamin D and C-reactive protein in asymptomatic adults (from the continuous National Health and Nutrition Examination Survey 2001 to 2006). *Am J Cardiol* 2012; 109:226–230. 21996139 10.1016/j.amjcard.2011.08.032

[47] Reyman M Verrijn Stuart AA van Summeren M Vitamin D deficiency in childhood obesity is associated with high levels of circulating inflammatory mediators, and low insulin sensitivity. *Int J Obes* 2014; 38:46–52. 10.1038/ijo.2013.75 23736361

[48] Ngo DT Sverdlov AL McNeil JJ Does vitamin D modulate asymmetric dimethylarginine and C-reactive protein concentrations? *Am J Med* 2010; 123:335–341. 20362753 10.1016/j.amjmed.2009.09.024

[49] Ahmad NS Khalid BA Luke DA Tocotrienol offers better protection than tocopherol from free radical-induced damage of rat bone. *Clin Exp Pharmacol Physiol* 2005; 32:761–770. 16173934 10.1111/j.1440-1681.2005.04264.x

[50] Nazrun AS Norazlina M Norliza M The anti-inflammatory role of vitamin e in prevention of osteoporosis. *Adv Pharmacol Sci* 2012; 2012:142702. 22162676 10.1155/2012/142702 PMC3226535

